# Machine learning-empowered *cis*-diol metabolic fingerprinting enables precise diagnosis of primary liver cancer†

**DOI:** 10.1039/d2sc05541d

**Published:** 2023-02-13

**Authors:** Pengfei Li, Shuxin Xu, Yanjie Han, Hui He, Zhen Liu

**Affiliations:** a State Key Laboratory of Analytical Chemistry for Life Science, School of Chemistry and Chemical Engineering, Nanjing University 163 Xianlin Avenue Nanjing 210023 China zhenliu@nju.edu.cn +86-25-8968-5639

## Abstract

*Cis*-diol metabolic reprogramming evolves during primary liver cancer (PLC) initiation and progression. However, owing to the low concentrations and highly structural heterogeneity of *cis*-diols *in vivo*, severe interference from complex biofluids and limited profiling coverage of existing methods, in-depth profiling of *cis*-diol metabolites and linking their specific changes with PLC remain challenging. Besides, due to the low specificity of widely used protein biomarkers, accurate classification of PLC from hepatitis still represents an unmet need in clinical diagnostics. Herein, to high-coverage profile *cis*-diols and explore the translational potential of them as biomarkers, a machine learning-empowered boronate affinity extraction-solvent evaporation assisted enrichment-mass spectrometry (MLE-BESE-MS) was developed. A single analytical platform integrated with multiple complementary functions, including pH-controlled boronate affinity extraction, solvent evaporation-assisted enrichment and nanoelectrospray ionization-based *cis*-diol identification, was constructed, which significantly improved the metabolite coverage. Meanwhile, by virtue of machine learning (principal components analysis, orthogonal partial least-squares discrimination analysis and random forest), collected *cis*-diols were statistically screened to extract efficient features for precise PLC diagnosis, and the results outperform the routinely used protein biomarker-based methods both in sensitivity (87.5% *vs.* less than 70%) and specificity (85.7% *vs. ca.* 80%). This machine learning-empowered integrated MS platform advanced the targeted metabolic analysis for early cancer diagnosis, rendering great promise for clinical translation.

## Abstract 2

A mass spectrometric platform was built for in-depth profiling of mutational landscape of *cis*-diol metabolites from the healthy to primary liver cancer (PLC) patients. This method enabled more precise PLC diagnosis than protein marker-based methods.

## Graphical abstract

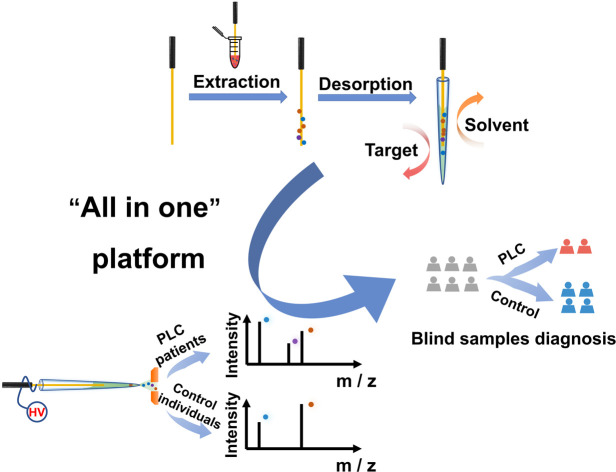

## Introduction

*Cis*-diol containing metabolites, including but not limited to carbohydrates, nucleosides, and nucleotides, are involved in a diverse range of biosynthetic and bioenergetic processes.^1–5^ Besides, they play vital roles in many essential physiological processes. The metabolic homeostasis of *cis*-diols is critical to maintain systematic functions such as energy storage and signal transduction.^4,6^ Dysregulation of *cis*-diol metabolism has been implicated in many tricky diseases such as cancers,^7–9^ diabetes,^10^ dyskeratosis congenital^11^ and Alzheimer's disease.^12^ Among them, the relationship between *cis*-diol alteration and primary liver cancer (PLC) particularly deserves more attention. Liver acts as the major machine in charge of whole-body metabolism and maintains metabolic homeostasis, and as a result, the onset and progression of PLC is frequently accompanied by rearrangements of metabolic pathways.^13^ In addition, three of four significant dysregulated metabolic pathways in PLC have been reported to be connected with *cis*-diols, *i.e.*, the hexosamine biosynthetic pathway, nucleotide synthesis pathway and glycolysis pathway.^14^ More importantly, despite the wide use of protein biomarker-based assays in early PLC diagnosis, limited effective biomarkers, low specificity and high-cost of corresponding antibodies still hindered further development.^15^ Even the routinely used clinical protein biomarker, alpha fetoprotein (AFP), still suffers from insufficient specificity (around 80%) and high false negative rate (35–59%) in real-world applications.^16,17^ Therefore, as a proof of concept, it is of paramount importance to explore the translational potential of *cis*-diol containing metabolites for precise PLC diagnosis. However, to the best of our knowledge, although metabolites are considered be more closely related to the phenotype of disease^18^ and have been applied in clinical studies for understanding disease progression,^19^ there is still an unmet demand for high-coverage screening of the mutational landscape of *cis*-diol metabolites from healthy individuals to hepatitis and PLC patients to provide a more holistic understanding of the pathologies of PLC underpinned by *cis*-diol metabolism.

Due to its strong qualitative ability and high-throughput capability, mass spectrometry (MS) has become the most powerful method for metabolic fingerprinting.^20–22^ However, the low concentrations and highly structural heterogeneity of *cis*-diols *in vivo* and complex biological matrix interference still severely limit further analysis of them,^23,24^ and thus rigorous pre-treatment procedures for the separation and enrichment of *cis*-diols from complex biofluids are usually indispensable prior to MS analysis. Liquid chromatography (LC) and capillary electrophoresis (CE) are leveraged by most traditional MS-based methods for separation.^25–27^ However, these separation techniques are also associated with apparent drawbacks. LC requires a long analysis time (*i.e.*, gradient elution/column equilibration),^28,29^ a large amount of sample while the column tends to be easily damaged by the high salt environment in biofluids.^30^ CE provides efficient separation with minimal band broadening for polar ionogenic metabolites, but its charge-dependent separation mechanism restricts the space of metabolites that can be separated.^31^ In contrast to conventional chromatography-coupled MS, direct infusion mass spectrometry (DI-MS), an ambient ionization approach involving the direct introduction of biological extracts into MS systems without any prior chromatographic separation,^32,33^ has been developed to couple with nanoelectrospray ionization (nESI) and successfully applied in serum analysis, such as paper spray^34^ and solid-phase microextraction (SPME)-based spray.^35^ It shows several advantages such as low sample volume requirement and high salt-cleaning efficiency. However, targets enriched onto the paper or SPME probe usually have much slower desorption kinetics, which limited the analytical sensitivity when combining those methods with MS. More importantly, multistep processing in those MS-based workflows often leads to significant sample loss, resulting in trade-offs between broad metabolome coverage and accessible sample size.^36^ To date, despite many advances in separation, enrichment and detection techniques, there is still no report of an integrated simple, sensitive and high-coverage DI-MS platform, hyphenated with the above three techniques, for low-volume serum *cis*-diols analysis, which is urgently needed to decipher the pivotal role of *cis*-diols as potential biomarkers in PLC-associated clinical studies.

A further challenge in linking specific *cis*-diol changes with PLC is the processing of MS big data to obtain the necessary accuracy. Machine learning provides a smart system with the ability to learn from big data and improve treatment methods in the healthcare sector. This technology has showed great potential in different clinical applications, such as imaging-guided surgical operation^37^ and untargeted metabolomics-based diagnosis;^38^ however, machine learning-empowered *cis*-diol analysis has not been explored yet until now.

**Fig. 1:**
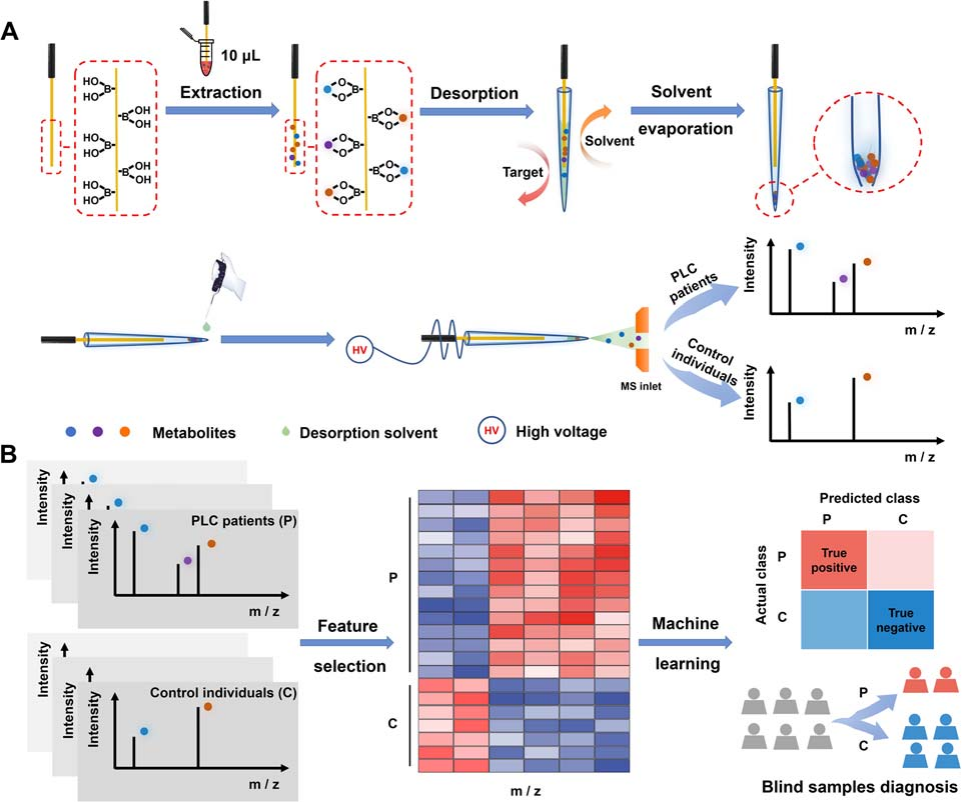
Schematic illustration of the solvent evaporation-assisted boronate affinity mass spectrometry and machine learning-based PLC diagnosis. (A) Workflow for the extraction of serum *cis*-diols metabolic patterns and the MS-based fingerprinting process. (B) Workflow for the feature selection and machine learning for blind sample diagnosis.

Herein, we report a machine learning-empowered boronate affinity extraction-solvent evaporation assisted enrichment-mass spectrometry (MLE-BESE-MS) for high-sensitivity serum *cis*-diol metabolic fingerprinting. Boronate affinity relies on reversible covalent interactions between boronic acids and *cis*-diol-containing compounds. In brief, boronic acids could form five or six-membered cyclic esters with *cis*-diols under high pH conditions (usually alkaline conditions) and the cyclic esters will dissociate when the environmental medium is changed to acidic conditions.^39^ This pH-controlled capture and release property made boronate affinity materials (BAMs) promising sorbents for the enrichment of *cis*-diols.^40,41^ However, real-world applications of BAMs were often severely hampered by non-biocompatible binding pH and weak affinity. Our group has been devoted to addressing the above issues by developing advanced BAMs for physiological pH binding and promoting their biomedical applications.^42–47^ In this study, a new integrated MS platform combined with boronate affinity extraction was constructed for separation, enrichment and sensitive metabolite identification. This platform endowed the integrated MS with appealing features, including high specificity, high signal-to-noise ratio detection capability, strong desalting-efficiency and low sample-volume required. What's more, combined with machine learning, we show that *cis*-diol features decoded by this integrated platform have translational potential for PLC diagnosis. The procedure of BESE-MS is illustrated in Fig. 1A. *Cis*-diol containing metabolites can be extracted effectively from 10 μL serum using a boronic acid-functionalized probe at physiological pH and meanwhile high-salt matrices can be removed simultaneously in the washing steps, and then the probe was inserted in the emitter for releasing *cis*-diols under 5 μL mass spectrometry compatible acid desorption solution. A solvent evaporation step with the help of an oven was applied in the desorption process. When compared with the traditional SPME probe-based methods of simultaneous desorption and MS detection, this process could effectively avoid sensitivity reduction caused by the low desorption kinetics of probe-extracted metabolites. Meanwhile, it made metabolites be concentrated in a smaller tip space, which is critical for generating high signal-to-noise signals in further processes. After that, a drop of desorption solvent was added to the tip of the emitter and about 20 nL desorption solvent was sucked into the emitter tip. In terms of our aim to address the trade-offs between broad metabolome coverage and low-volume sample size, a non-contact mode in nESI for induced ionization was applied.^48,49^ To be specific, a high voltage was applied to the probe while the probe was kept out of contact with the above desorption solution. Therefore, this ion source could be viewed as a capacitor formed with the probe/air/desorption solution in equivalent circuit and pulsed ions would be generated for spraying. Such pulsed nESI is highly compatible with a pulsed mass analyzer orbitrap,^49^ which could achieve high sample economy and make this MS method straightforward and practical for the analysis of complex biological fluids. Fig. 1B illustrates that the serum metabolic fingerprints can be leveraged with machine learning effectively for the early diagnosis of PLC, making this “all in one” platform not only a powerful tool in personalized diagnostics but also an important asset with great potential for multiple real-world applications.

## Results and discussion

### Synthesis and characterization of boronate affinity probes

**Fig. 2:**
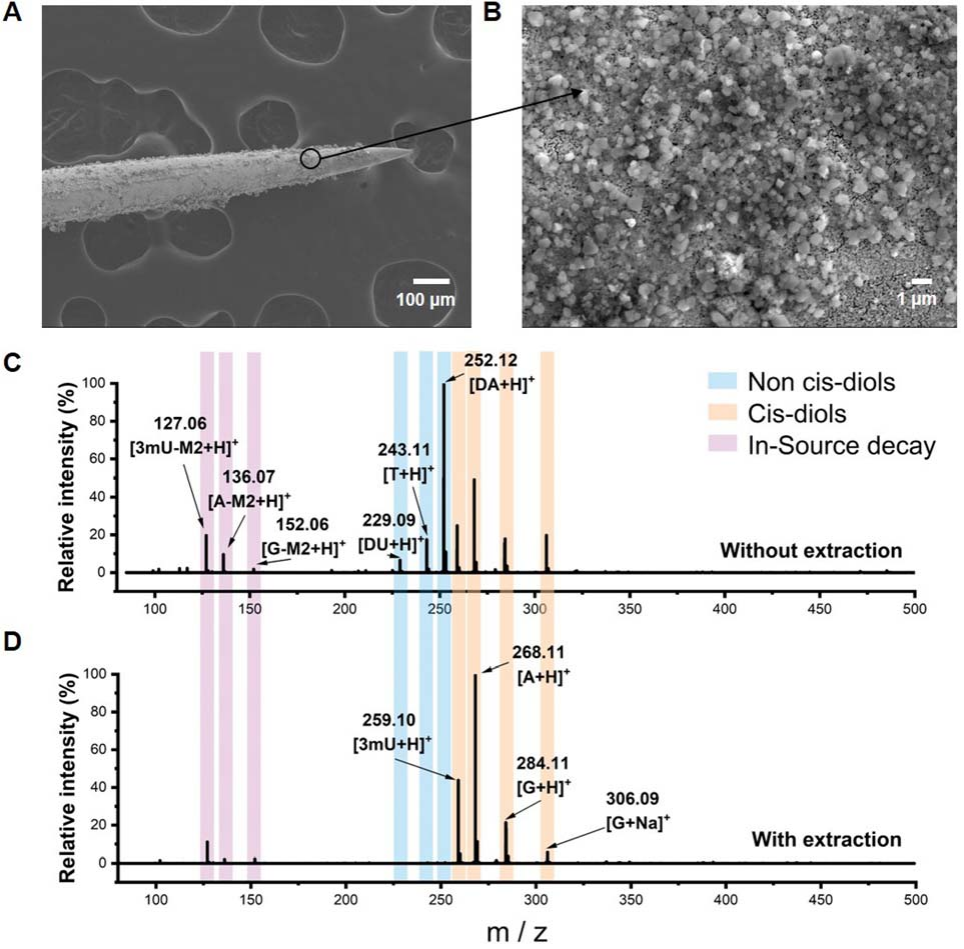
Characterization of the boronate affinity probe. (A) And (B) SEM characterization of the boronate affinity probe. (C) MS spectra of direct analysis of a mixture containing equal concentrations of standard adenosine, guanosine, 3-methyluridine, 2′-deoxyadenosine, 2′-deoxyuridine and thymidine. (D) MS spectra of compounds extracted using the boronate affinity probe from the above solution.

Boronate affinity extraction probes were first fabricated for specifically enriching *cis*-diols from biofluids. Commercial acupuncture needles were used as the substrate and coated with gold to enhance electrical conductivity, and then the probes were modified with aminopropyltriethoxysilane (APTES) and finally grafted with 2,4-difluoro-3-formylphenylboronic (DFFPBA). DFFPBA was selected as the affinity ligand because of its high boronate affinity toward *cis*-diol compounds.^46^ SEM characterization showed the rough surface of the probe (Fig. 2A and B). Such a rough surface provided a high specific surface area, which was beneficial for the extraction capacity. Energy dispersive spectrometer (EDS) characterization indicated the successful modification of gold and APTES on the probe (Fig. S1†). Because the relative atomic mass of boron was light and there is relatively low coverage of boron in the probe, the modification of DFFPBA could not be characterized by EDS. To overcome this issue, we designed another experiment for the validation of successful DFFPBA modification. The probe was immersed in 10 μL of ammonium bicarbonate buffer (50 mM, pH 8.5) containing adenosine and deoxyadenosine (1 mg mL^−1^ each) for extraction for 1 h and then eluted in 5 μL of 100 mM HAc. The same mixture solution without extraction and the extracts using the extraction probe were detected by MS. The results showed that the probe exhibited high affinity towards adenosine (a *cis*-diol containing compound) while almost no affinity towards deoxyadenosine (a non-*cis*-diol containing compound) (Fig. S2†), indicating successful DFFPBA modification on the probe. Besides, some recent studies have shown that boronic acid could interact with amino groups,^50^ while the results from Fig. S2† showed that the affinity of boronic acid to amino group is much lower than that to *cis*-diols, and thus the amino group-containing deoxyadenosine would be removed in washing steps, confirming the selectivity of the probe to *cis*-diols. Unlike fluorescence spectrometry,^51^ MS can detect multiple metabolites simultaneously without interfering with each other. For further investigating the multi-*cis*-diols binding capability of the probes and demonstrating the rationality of the combination of boronate affinity probes with MS, a more complex solution containing equal concentrations of standard adenosine (A), guanosine (G), 3-methyluridine (3mU), 2′-deoxyadenosine (DA), 2′-deoxyuridine (DU) and thymidine (T) was tested. The MS spectra of without extraction and extracts using the probe from the solution showed that the boronate-affinity probe enabled selective extraction of multiple *cis*-diols (Fig. 2C and D). In these experiments, the neutral loss of *cis*-diols due to the in-source decay was observed, which is in agreement with a previous report by others.^27^ For the convenience of *cis*-diol identification, the decay of the ribose moiety was annotated as *m*/*z* decreased by M2, and several other typical neutral losses of modified ribose moieties are summarized in Fig. S3.†

### Construction and performance evaluation of BESE-MS

**Fig. 3:**
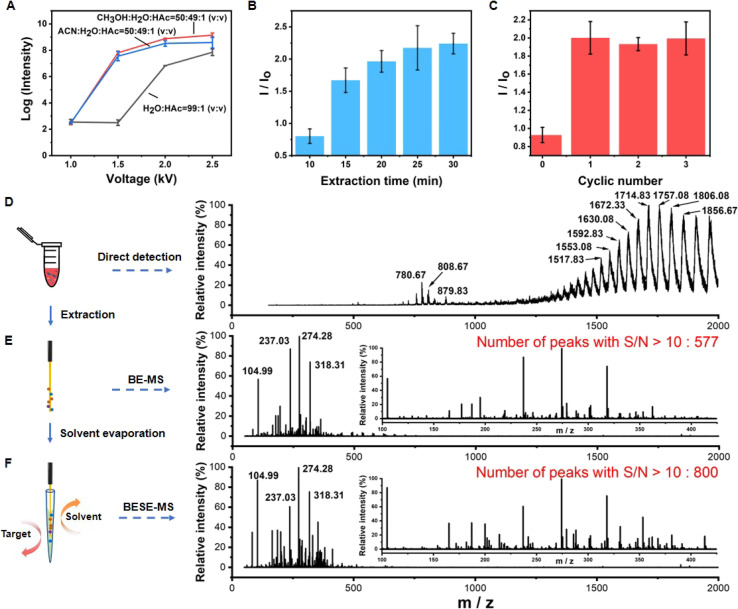
Construction and performance evaluation of BESE-MS. (A) Optimization of the voltage and desorption solvent for BESE-MS. (B) Optimization of extraction time (*n* = 3 independent repeats; mean ± s.d.). (C) Optimization of cycle number for the solvent evaporation step (*n* = 3 independent repeats; mean ± s.d.). (D) MS spectrum of 10-fold diluted serum. (E) MS spectra of B-MS. (F) MS spectra of BESE-MS.

Next, the BESE-MS method was constructed and optimized. The desorption solvent should not only easily break the boronate affinity interaction but also be compatible for MS analysis. Particularly, it should have relatively low surface tension so that it can be easily sprayed out. Meanwhile, the spray process should be initiated at a relatively low voltage for the sake of operation safety and energy saving. Hence, we optimized the desorption solvent and corresponding applied voltage first (Fig. 3A). The results showed that the desorption solvent of CH_3_OH : H_2_O : HAC = 50 : 49 : 1 (V : V) with 1.5 kV was suitable for further MS analysis. In addition, the extraction time and cycle number for solvent evaporation were optimized (Fig. 3B and C). We found that 25 min was appropriate and one cycle of solvent evaporation already met the detection needs, so these parameters were adopted in the follow-up experiments. Further, in order to investigate the sample loss in the extraction and desorption process, the analyte recovery was investigated. The results showed that the boronate affinity probes have high recovery in serum samples (80.34–103.85%) (Table S1†), which is well acceptable. Additionally, it is highly necessary to test the desalting ability of the boronate affinity probes due to the fact that there are high-concentration salts in serum, so we tested the extraction performance of the probes in different PBS solutions (Fig. S4†). The results showed that the probes could bind the target even in 10× PBS, which confirmed its high desalting ability. Besides, the reproducibility of solvent volume entering the emitter before nESI analysis was evaluated, because it is important for further quantitative analysis. The results showed that the volume of solvent is around 20 nL, and the relative standard deviation (RSD) value for three different emitters was 0.7% (Fig. S5†), which is much acceptable. What's more, BESE-MS showed several advantages when compared with a series of controlled trials. If 10-fold diluted serum was directly injected to the MS, human serum albumin (HSA) was dominant in the spectra and masked the information of metabolites (Fig. 3D) (the specific *m*/*z* and charges of HSA are summarized in Table S2†). When using the boronate affinity probe for extraction without the solvent evaporation step (Fig. 3E), the dominant HSA peaks in Fig. 3D completely disappeared while a lot of *cis*-diol containing metabolites were found. By virtue of the solvent evaporation method, the number of metabolites detectable at a high signal-to-noise ratio (S/N > 10) was apparently boosted, being 800 (Fig. 3F) as compared with 577 when the solvent evaporation step was absent (Fig. 3E). The mapping of the peak time and *m*/*z* values of detected metabolites resolved at the molecular species level explains why this method could perform better (Fig. S6†). After the solvent evaporation step, the metabolites could be concentrated in a smaller tip space and transferred together for detection, and thus high intensity signals could be detected in a less duration time (Fig. S6A†), resulting in a significantly increased S/N ratio in Fig. 3F. In contrast, in the method without the solvent evaporation step, the peak time of high intensity metabolites was dispersed and the longer duration time reduced their respective intensities (Fig. S6B†). Actually, glycoproteins, such as immunoglobin and transferrin which are highly abundant in serum, also belong to *cis*-diols, but they were hardly observed in the BESE-MS experiments. The absence of glycoproteins in our method is noteworthy, and we supposed that there are two reasons for this phenomenon: (1) in the direct immerse mode of SPME-based methods, the SPME probe can become quickly saturated with the most abundant analytes present in a given matrix (metabolites in serum),^52,53^ while other analytes (glycoproteins) remain unextracted;^54^ (2) even if a small amount of glycoproteins is extracted onto the probe, the detection of undesirable high molecular weight compounds will be easily interfered by matrix effects in the electrospray ionization source.^55^ The above comparison of BESE-MS with the direct serum detection method and BE-MS (boronate affinity extraction-mass spectrometry) indicates that our integrated method was not only effective in selective *cis*-diol analysis but also a sensitive and simple approach for general use.

### Qualitative and quantitative analysis of the targets of interest in biofluids

**Fig. 4:**
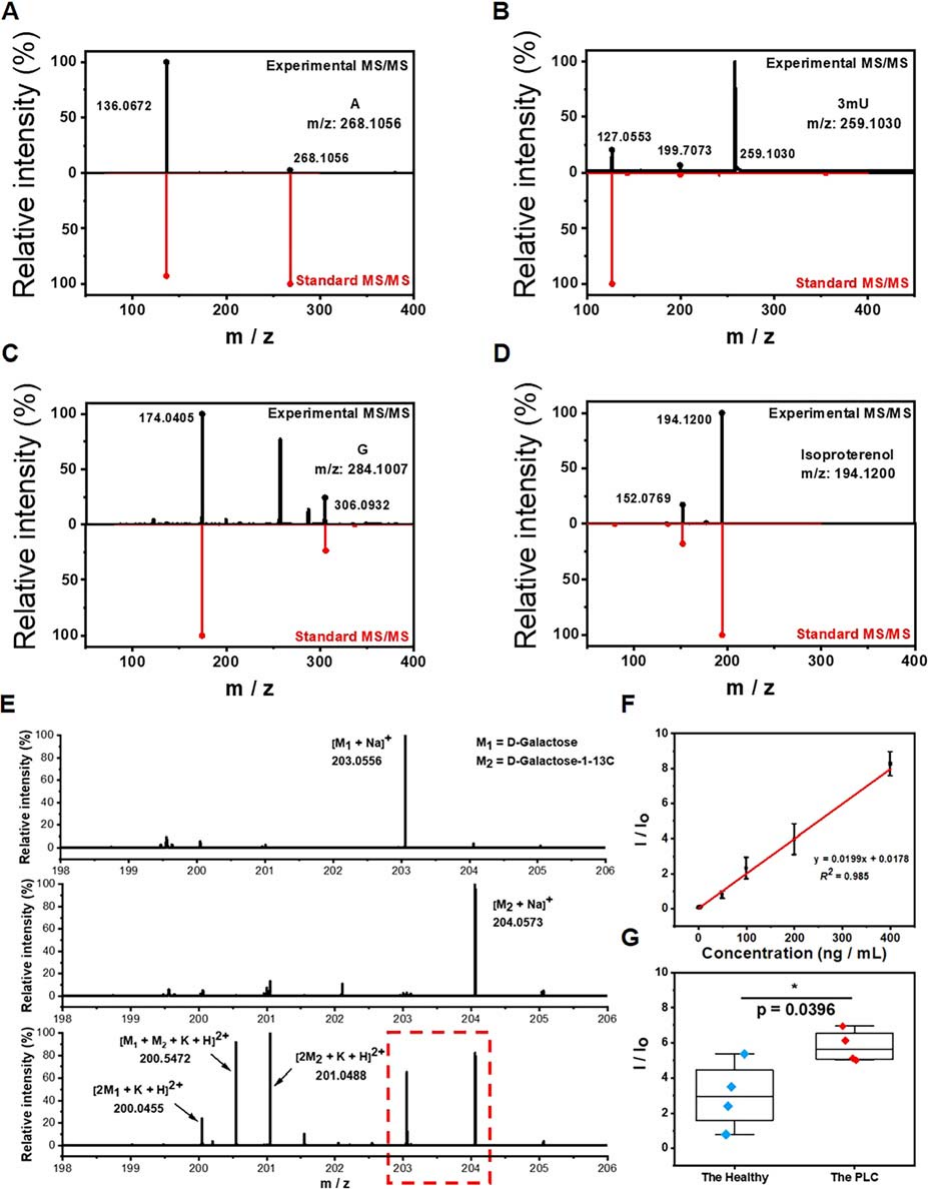
Qualitative and quantitative analysis of targets in serum. MS/MS spectra of adenosine (A), 3-methyluridine (B), guanosine (C) and isoproterenol (D). (E) MS spectra of the standard of d-galactose, standard of its isotope d-galactose-1-13C and equal concentration mixed solution of them. (F) Calibration curve for d-galactose with internal calibration. (G) The comparison of d-galactose in PLC patients and healthy individuals.

Precise identification and quantification of potential biomarkers in complex biological matrices are essential in clinical studies. Although we have showed the great selectivity of boronate affinity probes to *cis*-diols, it is still necessary to demonstrate the qualitative capability of BESE-MS for serum samples. Thus, we identified four *cis*-diol compounds from serum first based on accurate mass measurement with a high-resolution orbitrap mass analyzer and MS/MS experiments, and the identifications were further confirmed by chemical standards (Fig. 4A–D), indicating the remarkable practicability of this method in real-world applications. Besides, it should be noted that three of the four *cis* diols we selected are nucleosides (A, G and 3mU), because they play important roles in various biochemical processes. In addition, due to the lack of specific phosphorylases for modified nucleosides, 3mU cannot be recycled for synthesizing RNA, so it will be excreted in serum or urine and has been reported as an effective biomarker.^56^ Isoproterenol, a *cis*-diol drug for bronchial asthma and heart atrioventricular block, could be found in several donors and also selected here for identification, because it showed the great potential of our platform in pharmacokinetics analysis. To verify the quantification capability of the developed method, d-galactose was used as the target for quantitative analysis and its content difference between PLC patients and healthy individuals was investigated. d-galactose is an energy-providing nutrient and also a necessary basic substrate for the biosynthesis of many macromolecules in the body. Its alteration has been reported to be linked with cancers and therefore its detection could provide the knowledge of the basal metabolic level of bodies.^57^ We first tested the standard of d-galactose and its isotope d-galactose-1-13C at the same concentration (Fig. 4E). We found that their respective sodium ion adduct peaks were dominant in the spectra. Therefore, these peaks had potential as indicators of the concentration of d-galactose and its C13 isotope. When the two isotopes were mixed in equal volume and detected by MS, their respective sodium ion adduct peaks still kept unchanged; meanwhile, several dimerization cationic adduct peaks occurred, which indicates that the mass spectrum had enough high resolution to distinguish the target and its isotope peaks for quantitative analysis. The comparison of the MS spectra of the d-galactose isotope with the serum *cis*-diols showed that there was no peak overlap (Fig. 3F and 4E), confirming the further feasibility of using the C13 isotope of d-galactose as an internal standard (IS) in serum analysis. Next, we prepared several mixed solutions of d-galactose and its isotope with gradient concentration difference and used these to establish a calibration curve for the quantification of d-galactose in serum (Fig. 4F). The result showed that the ratio of target to IS obeyed a good linear relationship within the concentration range of 1 to 400 ng mL^−1^, and the LOD could be calculated as 0.3 ng mL^−1^. When no IS was employed, the linear relationship for calibration exhibited a worse correlation (Fig. S7†), pinpointing the great importance of introducing IS. Lastly, the difference of d-galactose in PLC patients and healthy individuals was investigated and the results showed that the concentration of d-galactose increased in cancer patients (Fig. 4G). This may be attributed to the abnormal expression of enzymes in cancer patients, resulting in the accumulation of the basic metabolites in the upstream of metabolic pathways.

### Clinical serum *cis*-diol fingerprinting and PLC diagnosis

**Fig. 5:**
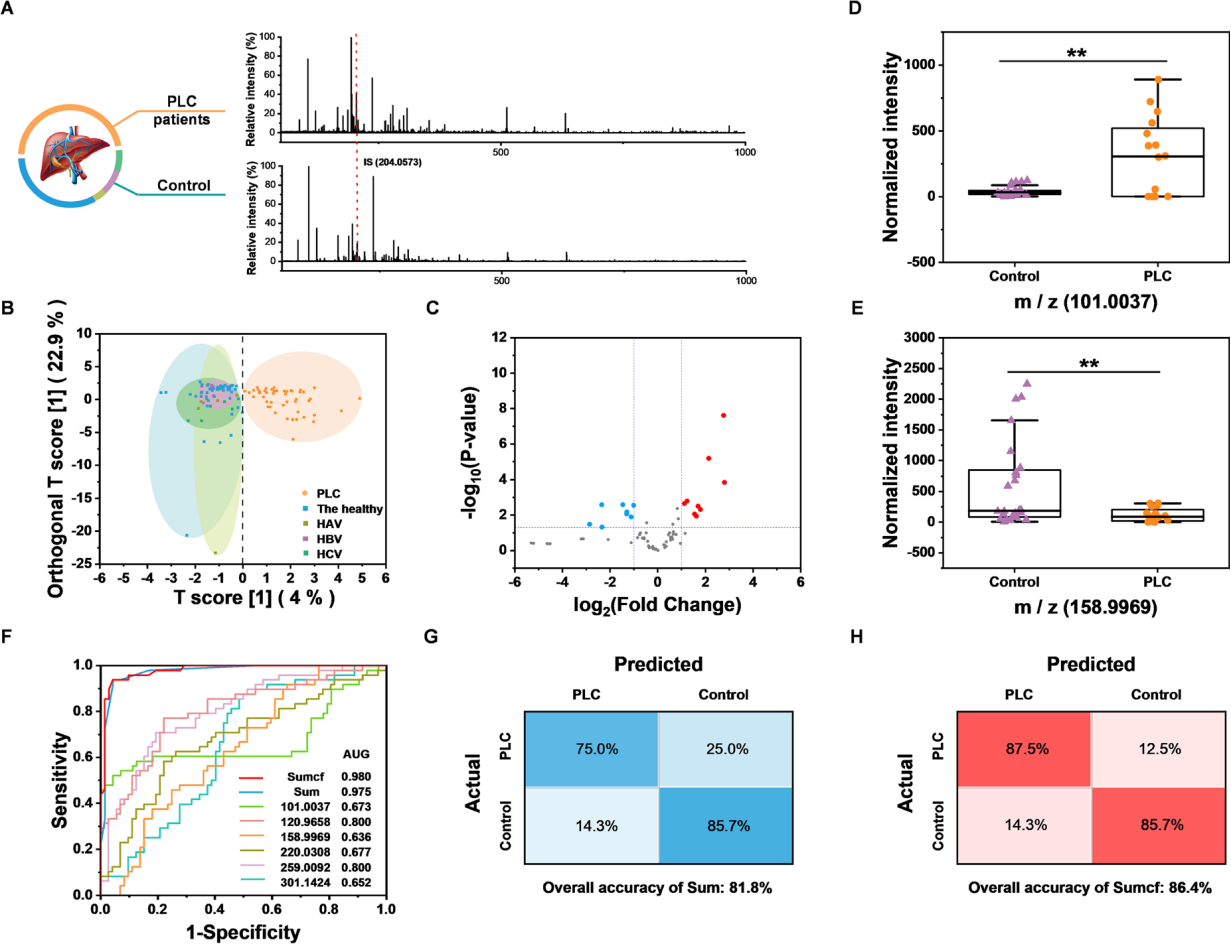
Clinical serum *cis*-diol fingerprinting and PLC diagnosis. (A) Raw MS spectra of PLC and control. (B) OPLS-DA analysis of the training dataset. (C) Volcano plot of the training dataset. (D) Comparison of the highest upregulated *m*/*z* values between PLC and control. (E) Comparison of the highest downregulated *m*/*z* values between PLC and control. (F) ROC of Sumcf, Sum and six single *m*/*z* features. (G) Confusion matrix for Sum. (H) Confusion matrix for Sumcf.

Having constructed and optimized the method, we further tested the feasibility of the BESE-MS platform for serum *cis*-diol fingerprinting and PLC diagnosis. A total of 62 serum samples were collected to extract metabolic patterns, including 24 PLC patients and 38 controls (24 healthy individuals, 3 hepatitis A virus (HAV) infected patients, 5 hepatitis B virus (HBV) infected patients and 6 hepatitis C virus (HCV) infected patients), and detailed information about these donors is summarized in Table S3.† To verify the PLC diagnosis capability, 40 samples (16 PLC patients and 24 controls) were used as the training dataset for building a diagnosis model, while the remaining 22 samples were used as the blind dataset for method validation. The typical raw MS spectra of PLC patients and controls are shown in Fig. 5A, and 200 highest peaks from every raw MS data of training samples were extracted for further analysis. Moreover, to demonstrate whether DFFPBA-modified probes played a key role in *cis*-diol profiling, probes without DFFPBA modification were used for serum extraction, while only the peaks from IS could be found in this control experiment (Fig. S8†), which demonstrated the practicability of our method in clinical serum *cis*-diol fingerprinting. Three machine learning algorithms were applied to the training dataset for distinguishing PLC patients from controls. Unsupervised principal component analysis (PCA) was used first but showed minor differentiation between the two groups (Fig. S9†). So, we further applied supervised algorithms to build classification models. Random forest (RF) was leveraged and the out of bag error was 0.075 (Fig. S10†), indicating the advanced diagnostic performance of the RF algorithm. The top ten significant features identified by RF are summarized in Fig. S11.† Then, the orthogonal partial least squares discriminant analysis (OPLS-DA) was used to differentiate the PLC patients from the healthy controls. The result showed that the two groups could be significantly separated based on extracted metabolite fingerprinting (Fig. 5B), confirming the potential of *cis*-diols as biomarkers. More importantly, because nearly 30% PLC patients are AFP negative (AFP < 25 ng mL^−1^) and AFP is elevated not only in PLC but also in many other diseases, such as viral hepatitis, traditional AFP-based assays fail to well meet the needs for clinical precise PLC diagnosis. Meanwhile, although some other biomarkers, such as extracellular vesicles, have also been reported for great performance in PLC diagnosis, further research on classification of PLC from viral hepatitis has not been carried out.^58^ Therefore, the OPLS-DA result that screened *cis*-diols could effectively distinguish the PLC from three kinds of viral hepatitis, which is really inspiring and also demonstrates its necessity for precise PLC diagnosis. Because the OPLS-DA algorithm showed much better performance than RF, it was chosen to extract features for further use, and the top ten features with the highest variable importance on projection (VIP) from OPLS-DA are summarized in Fig. S12.† The volcano plot was also used to find the difference between the two groups, and totally 9 upregulated as well as 8 downregulated metabolites were found (Fig. 5C); at the same time, detailed fold changes and *p* values from the volcano plot are summarized (Table S4†). In terms of the fold change and VIP value from OPLS-DA, we chose six *m*/*z* features as indicators to establish the diagnostic model, and the two metabolites with the highest up- and down-fold change are shown with a box plot for comparison of the PLC group and the control group (Fig. 5D and E), which demonstrated the potential of those metabolites for PLC diagnosis. In addition, the comparison of the other four *m*/*z* features between PLC and controls is shown in Fig. S13.† However, the receiver operating characteristic (ROC) curve showed that it was not appropriate to diagnose PLC with these *m*/*z* features respectively, because of the low area under curve (AUC) (Fig. 5F). Hence, we introduced a new input parameter Sum, which is the sum of normalized and scaled intensity of the above six values. The AUC of Sum in the training dataset was calculated to be 0.975 (Fig. 5F), which demonstrated the high sensitivity and specificity of Sum to distinguish PLC cases from controls in the training dataset. From the ROC curve, the max Youden index value could be calculated as 0.896, which indicated the effectiveness of Sum as a diagnostic marker; in addition, this value was selected as an optimal cut-off point to distinguish controls from PLC cases. When applying Sum in the blind dataset, the overall accuracy reached 81.8% (Fig. 5G), which is well acceptable. To improve the overall accuracy, we further introduced another parameter, Sumcf, in which the VIP values were introduced to multiply six *m*/*z* values as the correction factor on the basis of the Sum. The AUC of Sumcf increased to 0.980 (Fig. 5F) and the overall accuracy increased to 86.4%. At the same time, the sensitivity and specificity in the confusion matrix reached 87.5% and 85.7% for the blind sample test (Fig. 5H), which are much more precise than protein-based diagnosis. Those results confirmed the successful optimization and demonstrated the translational potential of our method for using *cis*-diol metabolites to precisely diagnose PLC. However, it should be noted that existing results are limited by datasets and algorithms. For further evaluating the accuracy of the built model in a larger human cohort, false discovery rate (FDR) should be calculated. The calculation results are summarized in Table S5† and it demonstrates that *m*/*z* of 101.0037 is the most potential biomarker candidate for further clinical analysis. And we foresee further development in this method by recruiting more donors and applying more advanced algorithms.

## Conclusions

Primary liver cancer is the fourth most common cause of cancer-related death worldwide, and >80% of PLC cases occur in medical resource-constrained countries, leading to the steady rise of the mortality rate of PLC cases.^59^ Hence, developing early PLC diagnostical methods can not only effectively improve survival but also practically decrease the medical burden of resource-limited regions. However, current gold standard methods, the complex combination of imaging and histopathological examinations of tissue, may lead to an increased risk of lesions to patients and often require expensive instruments.^58^ In comparison, blood-based liquid biopsies could offer a non-invasive and economic approach. Despite the wide use of protein biomarker-based biopsies, limited effective biomarker candidates, low specificity and corresponding high-cost of antibodies still hindered further development.^15^ Therefore, developing metabolite biomarker-based biopsies is an appealing alternative for precise PLC early diagnosis.

*Cis*-diols represent an important class of metabolites and have been reported to be connected with PLC. However, the correlation between *cis*-diols and PLC is not well explored, because the analysis of *cis*-diols has been largely restricted by coverage, accessible sample size and matrix interference. What's more, although several studies have evaluated the potential of urine *cis*-diols for the diagnosis of PLC,^9^ few have examined serum *cis*-diols.^60,61^ In this work, we first developed an integrated machine learning-empowered boronate affinity extraction-solvent evaporation assisted enrichment-mass spectrometric platform for low-volume serum *cis*-diol analysis and PLC diagnosis. We demonstrated that boronic acid-functionalized probes could selectively capture *cis*-diol containing metabolites from serum and couple with the solvent evaporation step and nESI for selective, simple and sensitive analysis. The combination of orthogonal separation and enrichment steps at a single analytical platform showed enhanced ability in metabolic coverage. And the machine learning-empowered *cis*-diol fingerprinting results demonstrated that our method could enable more precise PLC diagnosis than existing protein biomarker-based methods. What's more, this method not only performed well with high specificity, high sensitivity, strong desalting-efficiency and low sample-volume required for *cis*-diol analysis, but also have potential to be leveraged for another significant targeted metabolite fingerprinting with customized probes. Hence, this machine learning-empowered MS platform holds great promise for metabolic analysis in multiple areas, including targeted metabolomics, disease biomarker screening and diagnosis, and unique metabolic pathway analysis.

## Data availability

The data that support the findings of this study are available from the corresponding author upon reasonable request.

## Author contributions

Z. L. conceived the idea. P. L. carried out the materials synthesis, MS experiments and data analysis. S. X. and Y. H. helped with the sample collection. H. H. participated in the discussion. Z. L. and P. L. wrote the manuscript.

## Conflicts of interest

The authors declare no competing interests.

## Supplementary Material

SC-014-D2SC05541D-s001

SC-014-D2SC05541D-s002
