# Supplementary material for: Machine learning-empowered cis-diol metabolic fingerprinting enables precise diagnosis of primary liver cancer
Source: Chem Sci. 2023 Feb 13;14(10):2553–61. doi: 10.1039/d2sc05541d (PMC9993839; doi:10.1039/d2sc05541d)
Supplement: SC-014-D2SC05541D-s001 [file SC-014-D2SC05541D-s001.pdf]

Electronic Supplementary Material (ESI) for Chemical Science.
This journal is © The Royal Society of Chemistry 2023

Electronic Supplementary Information for

Machine Learning-Empowered Cis-diol Metabolic Fingerprinting Enables Precise Diagnosis of Primary Liver Cancer

Pengfei Li, Shuxin Xu, Yanjie Han, Hui He, and Zhen Liu*

*Corresponding author. Email: zhenliu@nju.edu.cn

This PDF file includes:

Supplementary Text
Figs. S1 to S13
Tables S1 to S6
Reference

Supplementary Text

Materials

Adenosine (Molecular weight (MW)=267.24), thymidine (MW=242.23) and 2,4-difluoro-3-formyl-phenylboronic acid (DFFPBA) were all purchased from Sigma-Aldrich (St. Louis, MO, USA). Commercial acupuncture needles, guanosine (MW=283.24), isoproterenol hydrochloride, 3-methyluridine (MW=258.23) and 2'-deoxyadenosine (MW=251.24) were purchased from Liangwei Biotechnology (Nanjing, China). 2'-deoxyuridine (MW=230.21) was purchased from Topscience Co. Ltd. (Shanghai, China). Glacial acetic acid (HAc) was obtained from Sinopharm Chemical Reagent (Shanghai, China). Aminopropyltriethoxysilane (APTES, 98%), galactose (MW=180.16) and D-galactose-1-13C (MW=181.16) were purchased from J&K scientific (Shanghai, China). Chloroauric acid ($HAuCl_4·4H_2O$), potassium bicarbonate, glucose, anhydrous ethanol and ammonium bicarbonate were purchased from Nanjing Reagent Company (Nanjing, Chain). Methanol and acetonitrile (ACN) were purchased from Shanghai Macklin Biochemical (Shanghai, China). 10X, 1X and 0.1X phosphate-buffered saline (PBS) solution were from Keygen Biotech (Nanjing, China). Glass micropipette of 0.58 mm i.d. and 1.0 mm o.d. were purchased from DL Naturegene Life Sciences (Shanghai, China). Acupuncture needles of 0.16 mm diameter and 40 mm length were purchased from Aikang Medical Company (Changchun, China). Serum samples of 10 HCC patients and 4 healthy individuals were obtained from Jiangsu Province Hospital (Nanjing, China) and approved by the Institutional Ethics Committee of Jiangsu Province Hospital. Remaining serum samples of HCC patients, healthy individuals, 3 HAV infected patients, 5 HBV infected patients and 6 HCV infected patients were obtained from Kaifeng Central Hospital (Kaifeng, China) and approved by the Institutional Ethics Committee of Kaifeng Central Hospital. Unless otherwise specified, all other reagents used were of analytical grade or higher purity. Water used in all the experiments was purified with a Milli-Q Advantage A10 ultrapure water purification system (Millipore, Milford, MA, USA).

Mass spectrometer

Thermo fisher LTQ-Orbitrap XL. All MS characterizations were carried out on positive-ion mode. Easy spray ion source parameters were as follows: spray voltage of 1.50 kV, capillary temperature of 80 °C and tube lens at 110 V. FTMS analyzer parameters were as follows: resolution of 30000, max inject time of 100 ms.

Methods

Preparation of glass micropipette.

Borosilicate glass capillaries were pulled by P-2000 pipette puller (Sutter Instrument, Novato, CA, USA) to prepare emitters for MS analysis. Parameters of P2000 were optimized as follows: HEAT = 500, FIL = 3, VEL = 25, DEL = 180, PUL = 200.

Preparation of boronic acid-functionalized extraction probes

Acupuncture needles were immersed in a mixed solution (12 mM $HAuCl_4$, 0.5 M $KHCO_3$ and 25 mM glucose) for 5 h at 55 ℃ (air bath), next washed with water for three times and dried at room temperature. The obtained Au-coated probes were immersed in 4 % APTES ethanol solution for 5 h and washed with ethanol for three times. After that, the probes were

immersed in 100 mL mixed solution of 5 mg/mL sodium cyanoborohydride and 5 mg/mL DFFPBA for 24 h at room temperature, the probes were washed with ethanol for three times and dried at room temperature for further use.

Characterization of boronic acid-functionalized extraction probes.
A boronic acid-functionalized probe was immersed in 1) 10 μL ammonium bicarbonate buffer (50 mM, pH 8.5) containing adenosine and deoxyadenosine (1 mg/mL each) or 2) 10 μL ammonium bicarbonate buffer (50 mM, pH 8.5) containing 1 mg/mL standard of adenosine, guanosine, 3-methyluridine, 2'-deoxyadenosine, 2'-deoxyuridine and thymidine for 1 h. After that, the probe was rinsed with ammonium bicarbonate buffer (50 mM, pH 8.5) for three times each and inserted into a glass micropipette preloaded with 5 μL 100 mM HAc solution for 1 h, then the glass micropipette was used as an emitter for MS analysis.

Development of boronate affinity extraction-solvent evaporation assisted enrichment-mass spectrometry
To develop the method, three aspects were optimized: 1) the solvent for desorption and voltage used for nESI, 2) the extraction time, and 3) the solvent evaporation cyclic number for desorption.

Optimization of the desorption solvent and voltage used in nESI
Probes were immersed in 10 μL ammonium bicarbonate buffer (50 mM, pH 8.5) containing 1 mg/mL adenosine for 1 h. Then the probes were washed with ammonium bicarbonate buffer for three times. After that, probes were inserted into the glass micropipettes preloaded with 5 μL different desorption solutions (CH_3OH: H_2O: HAC = 50:49:1 (V: V), ACN: H_2O: HAC = 50:49:1 (V: V), H_2O: HAC = 99:1 (V: V)). Next, the probes-inserted micropipettes were placed in vacuum oven and temperature was set as 40 °C for solvent evaporation. Above desorption operation was repeated three times. Subsequently, the glass micropipettes were used as emitters. A drop of corresponding desorption solvent was added to the tip of emitters and about 20 nL desorption solvent was sucked into the emitter tip for further nESI analysis. A high voltage was applied to the probe through the copper wire. Single ion mode (SIM) within 6 m/z range was used to record the mass spectra. The measurement was repeated three times each.

Optimization of the extraction time
Probes were immersed in 10 μL ammonium bicarbonate buffer (50 mM, pH 8.5) containing 1 μg/mL galactose for 10, 15, 20, 25 or 30 min, then probes were washed with ammonium bicarbonate buffer for three times. After that, the procedure is almost the same as optimization method described above except the following difference. The desorption solution that preloaded in glass micropipettes was prepared as follow: CH_3OH: H_2O: HAC = 50:49:1 (V: V). And the solvent added to the tip of emitters for nESI analysis was above desorption solution containing 50 ng/mL D-galactose-1-13C.

Optimization of the solvent evaporation cyclic number for desorption
Probes were immersed in 10 μL ammonium bicarbonate buffer (50 mM, pH 8.5) containing 1 μg/mL galactose for 25 min, then probes were washed with ammonium bicarbonate buffer for three times. After that, the procedure is almost the same as optimization of extraction

time described above except the following difference. The desorption process in oven were operated with 0, 1, 2 or 3 times.

Characterization of analyte recovery

10 μL of healthy human serum was used to measured its galactose content by BESE-MS (initial content). Then 50 μL of the same human serum and 10 μL 500 ng/mL of galactose aqueous solution were mixed. After fully shaking and mixing, three 10 μL of sample were taken out from above mixed solution. Then their galactose contents were measured by BESE-MS method (Contents after adding standard).

Characterization of desalting ability of BESE-MS.

The probes were immersed in 10 μL 10 X, 1 X and 0.1 X PBS containing adenosine and deoxyadenosine (1 mg/mL each) for 25 min, then the probes were washed with ammonium bicarbonate buffer for three times. After that, the probes were inserted into the glass micropipettes preloaded with 5 μL desorption solution (CH_3OH: H_2O: HAC = 50:49:1 (V: V)) at 40 °C in vacuum oven for solvent evaporation. Next, the glass micropipettes were used as emitters. A drop of desorption solvent was added to the tip of emitters and about 20 nL desorption solvent was sucked into the emitter tip for further nESI analysis. At the meantime, the 10 X, 1 X and 0.1 X PBS containing 1mg/mL adenosine and 1mg/mL deoxyadenosine were loaded 5 μL each into another micropipettes for control MS analysis.

Serum Cis-diols fingerprinting

Probes were immersed in 10 μL serum for 25 min, then probes were washed with ammonium bicarbonate buffer for three times. After that, probes were inserted into the glass micropipettes preloaded with 5 μL desorption solutions (CH_3OH: H_2O: HAC = 50:49:1 (V: V)). Next the probes-inserted micropipettes were place in vacuum oven and temperature was set as 40 °C for solvent evaporation. Subsequently, the glass micropipettes were used as emitters. A drop of desorption solvent containing 50 ng/mL D-galactose-1-13C was added to the tip of emitters and about 20 nL desorption solvent was sucked into the emitter tip for further nESI analysis. 1.5 kV high voltage was applied to the probe through the copper wire, and the measurement was repeated three times each. And the quality control (QC) experiments were carried out according to a previously reported method.[1] Specifically, the pooled QC sample was formed by completely mixing 10 μL of each serum samples. Then each 10 μL of above QC sample was added separately into different centrifuge tube. The remaining preparation process were all the same as that to test samples. Triplicate pooled QCs were run every six test samples to monitor the technical quality of the results.

Statistical analysis

The metabolite features were extracted with the help of MS-DIAL.[2] Accurate m/z values from online databases (METLIN: metlin.scripps.edu; HMDB: www.hmdb.ca) were referred for metabolite peak assignments, in addition, the relative error was set as 15 ppm and $[M+H]^+$, $[M+Na]^+$, $[M+K]^+$, $[M+H-H_2O]^+$ were used for setting adduct types.[3, 4] All peaks from one sample spectra were sequenced by intensity and top 200 peaks were selected as fingerprint for further analysis. 200 highest peaks were extracted from each raw MS data of serum samples with the help of Xcalibur. Following alignment, a series of filters were applied: (1) prevalence filtering by only retaining peaks present in at least 80% of the biological samples,

(2) filtering peaks with 30% missing values or not detected in the QC sample. After that, the dataset was subjected to MetaboAnalyst for missing values imputation (missing values will be replaced by 1/5 of min positive values of their corresponding variables), normalization (against the IS D-galactose-1-13C), G-log transformation, autoscaling, and statistical analysis (Volcano plot analysis, PCA analysis, OPLS-DA analysis and random forest analysis).[5-7] Then, the cis-diol changes from different machine learning algorithms were comprehensively compared to find the potential biomarkers. The ROC curve was calculated in SPSS statistics 26, and then data were exported for drawing in origin.

Safety Considerations

Prevention of injuries and exposures should be noted for working with HBV and HCV samples. Technicians who handle blood are suggested to be vaccinated. All blood must be handled with gloves. Eye wear and protective clothing are needed. All plastic and glassware contaminated with serum should be placed in a plastic autoclave bag for disposal. These bags should be kept in appropriate containers until sealed and autoclaved. All work surfaces should be wiped down with 10% bleach solution.[8]

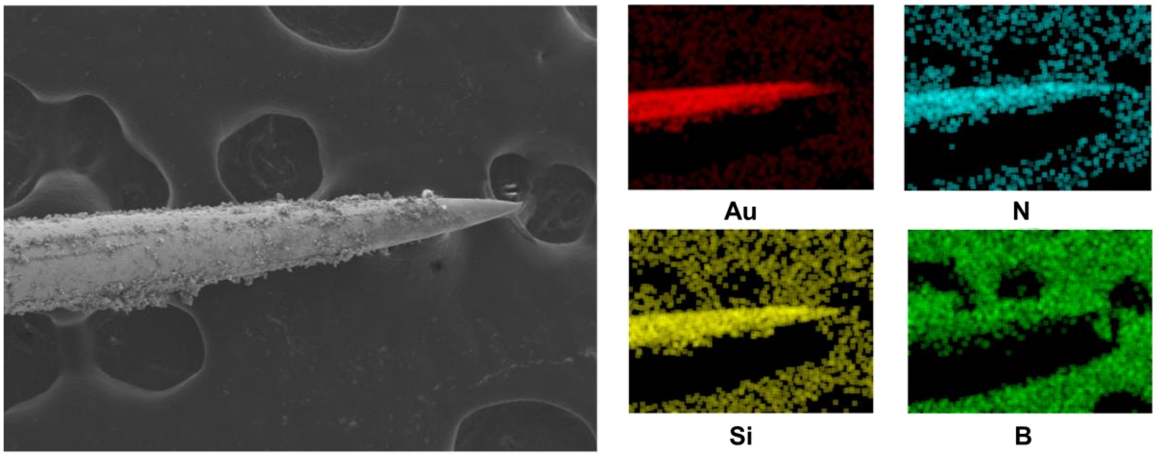

Fig. S1.

Energy Dispersive Spectroscopy of a boronate affinity probe.

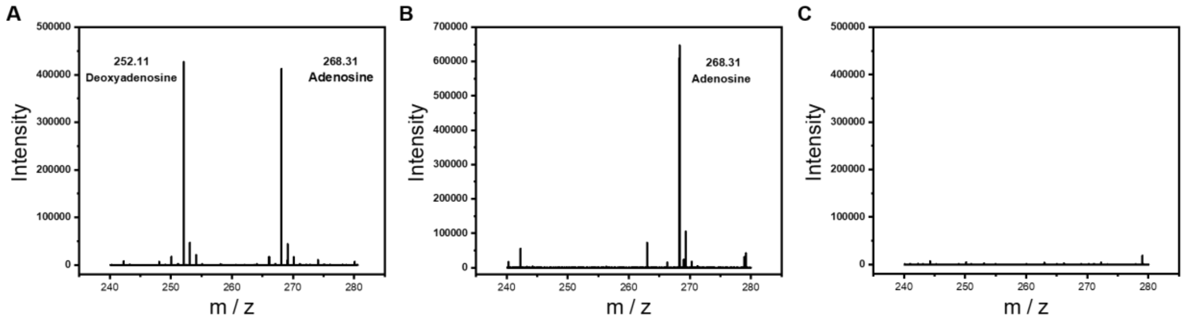

Fig. S2.

Characterization of the selectivity of a boronate affinity probe. MS spectra for direct analysis of (A) a solution containing adenosine and deoxyadenosine (1 mg/mL each), (B) analyte extracted from solution containing adenosine and deoxyadenosine (1 mg/mL each) by a boronate affinity probe and (C) elution from an unmodified probe, which was used as control probe to extract in solution containing adenosine and deoxyadenosine (1 mg/mL each).

.

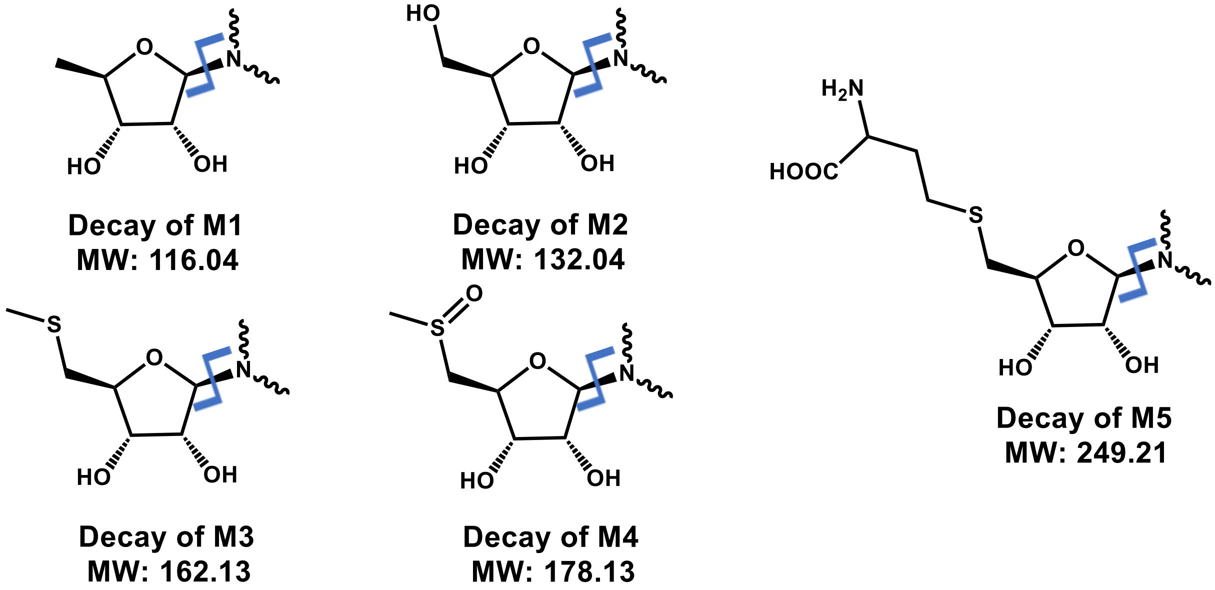

Fig. S3.

Illustration of neutral losses of typical cis-diols (MW: molecular weight of neutral losses).

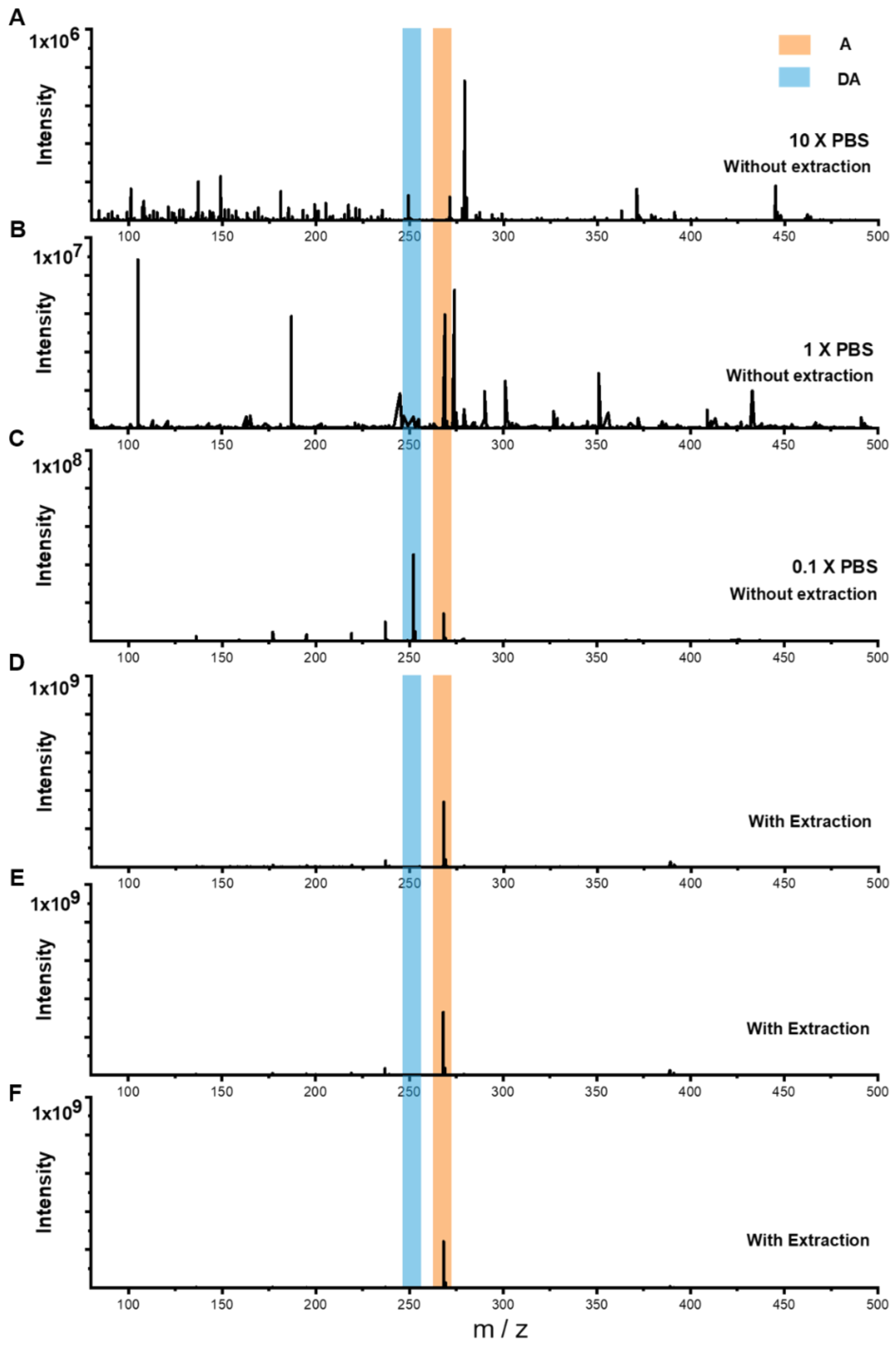

Fig. S4.

Characterization of the desalting ability of BESE-MS. MS spectra for direct analysis of (A) 10 X, (B) 1 X and (C) 0.1 X PBS solution containing adenosine and deoxyadenosine (1 mg/mL each); MS spectra of analyte extracted from (D) 10 X, (E) 1 X and (F) 0.1 X PBS solution containing adenosine and deoxyadenosine (1 mg/mL each) by boronate affinity probes. (Intensity of adenosine from A to F: 325; 5.96×10^{6}; 1.43×10^{7}; 3.40×10^{8}; 3.29×10^{8}; 2.43×10^{8}.)

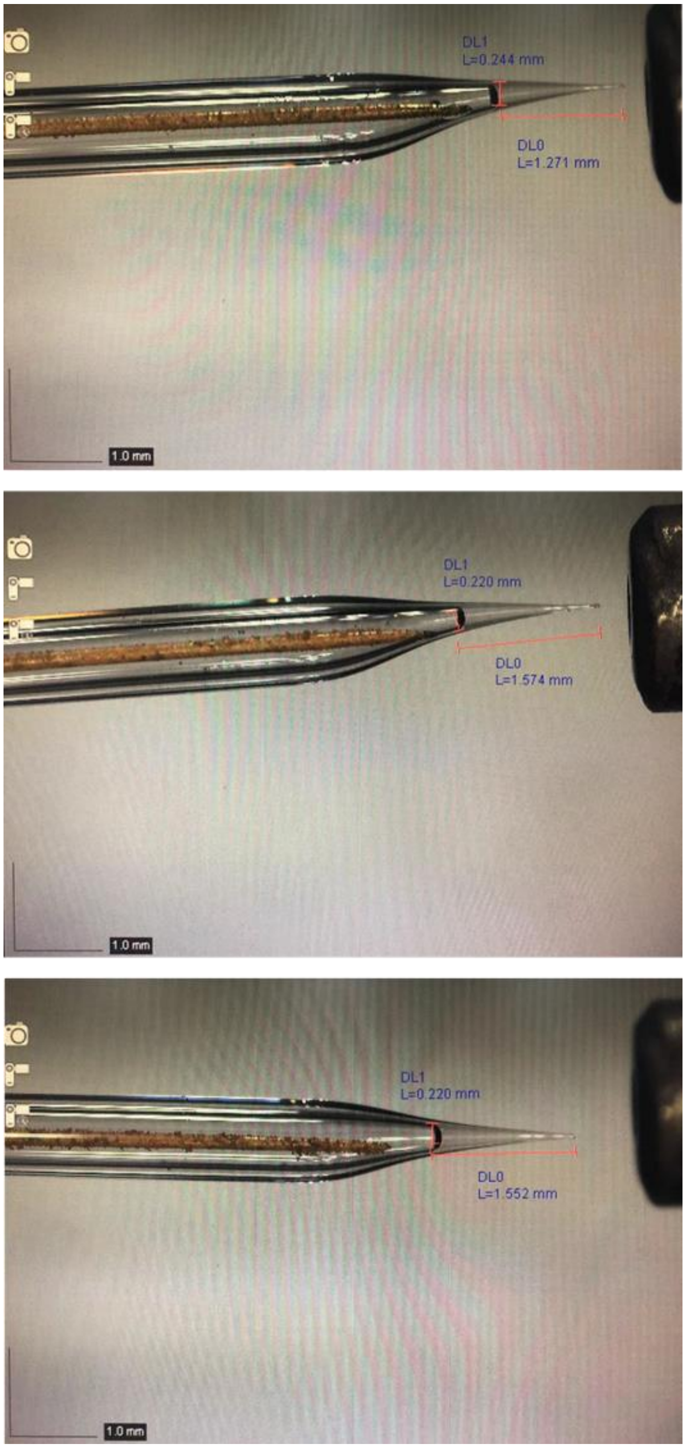

Fig. S5.

Characterization of solvent volume entered the emitter before nESI analysis.

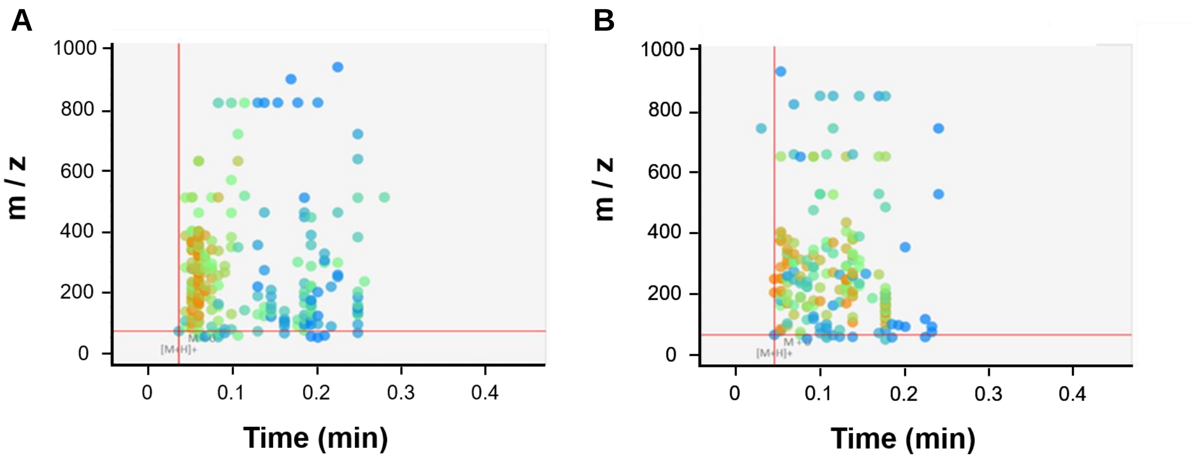

Fig. S6.

Mapping of time and m/z values of detected metabolites from total ion chromatogram. (A) from BESE-MS; and (B) from BE-MS. The color of the dots represents metabolites abundance: orange, green and blue represent abundance from high to low respectively.

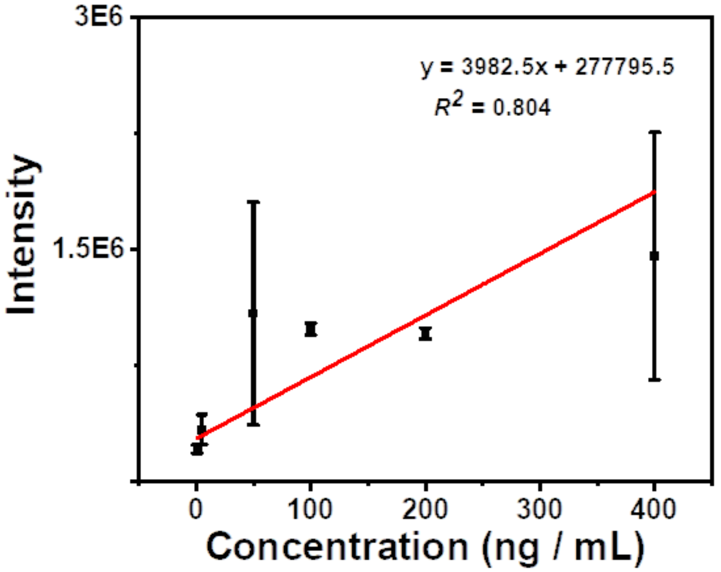

Fig. S7.
The linear relationship of m/z signal at 203.0556 with the concentration of D-galactose.

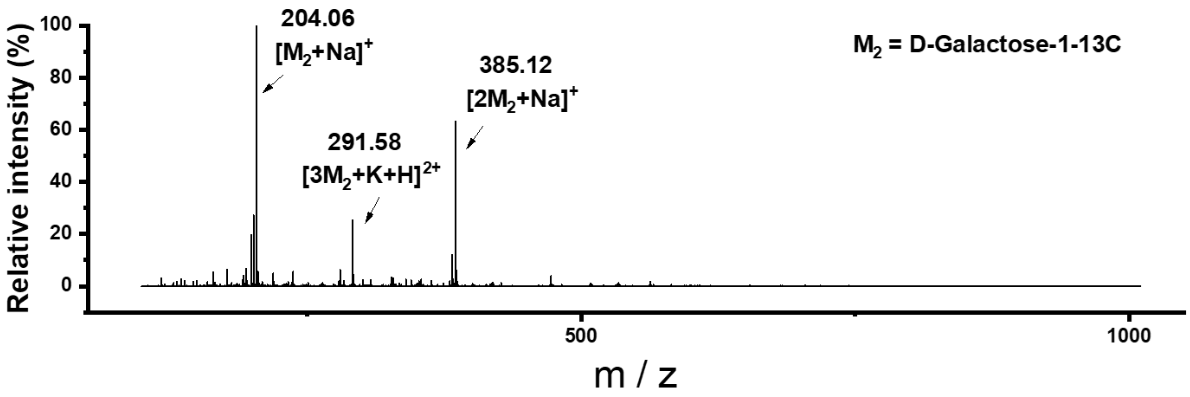

Fig. S8.

MS spectrum for serum analysis with analytes extracted by probes without DFFPBA modification.

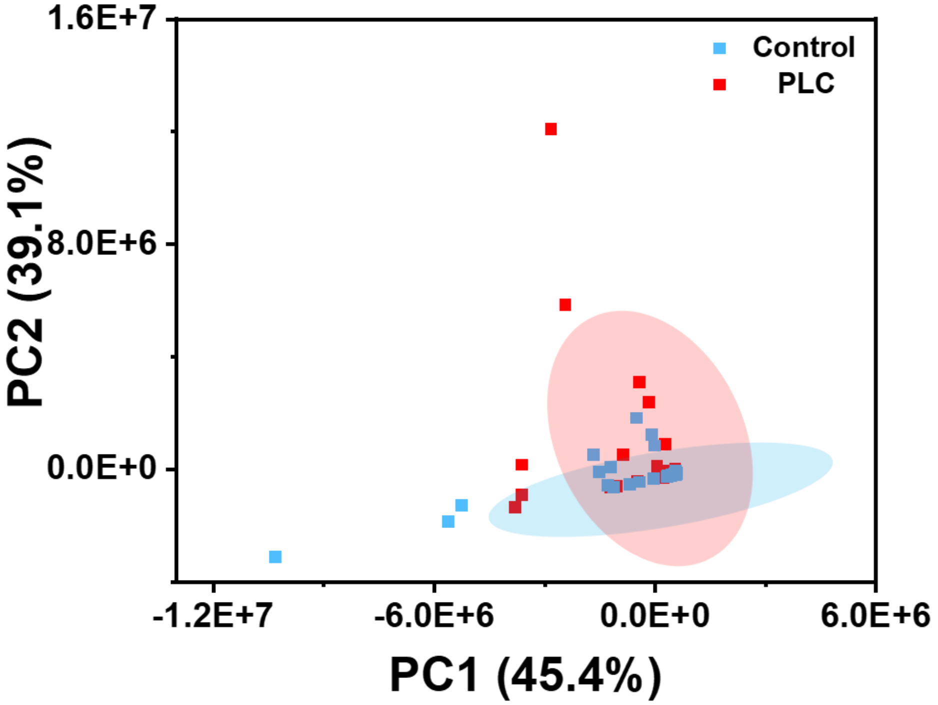

Fig. S9.

PCA analysis of training dataset. The results showed unsupervised analysis can only showed minor differentiation between PLC and control in training dataset.

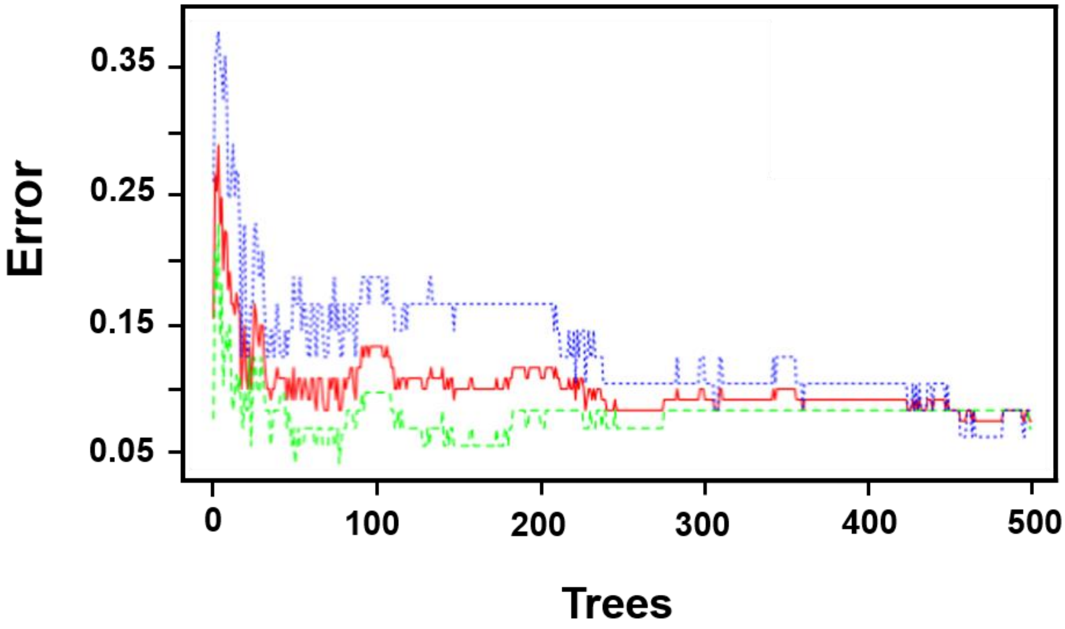

Fig. S10.

Cumulative error rates by Random Forest classification. The overall error rate is shown as the red line; the blue and green lines represent the error rates for the PLC and the healthy. The out of bag error (for red line) is 0.075.

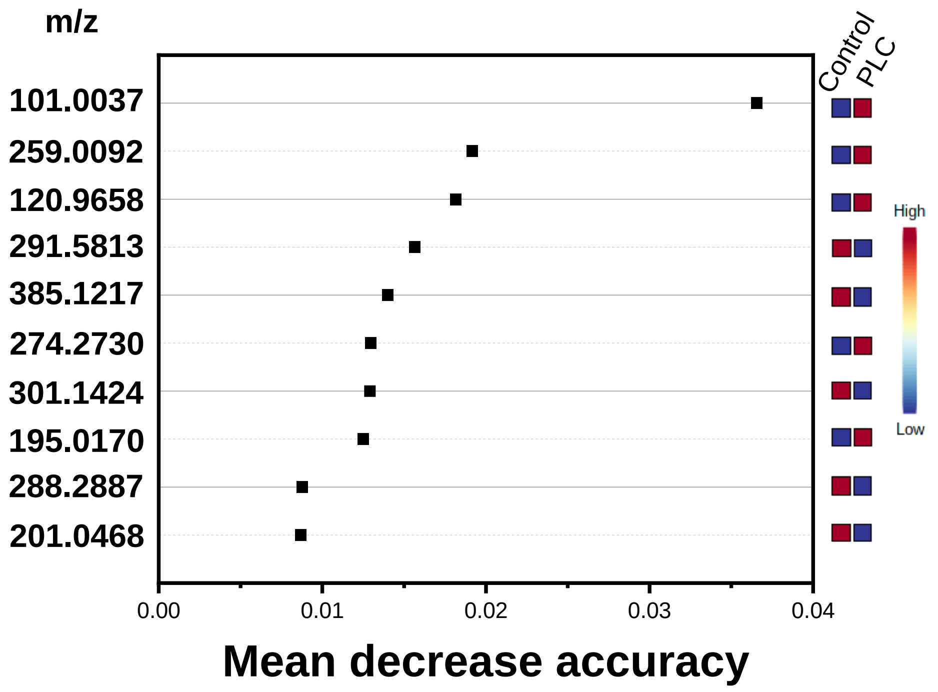

Fig. S11.

Significant features identified by Random Forest. The features are ranked by the mean decrease in classification accuracy when they are permuted.

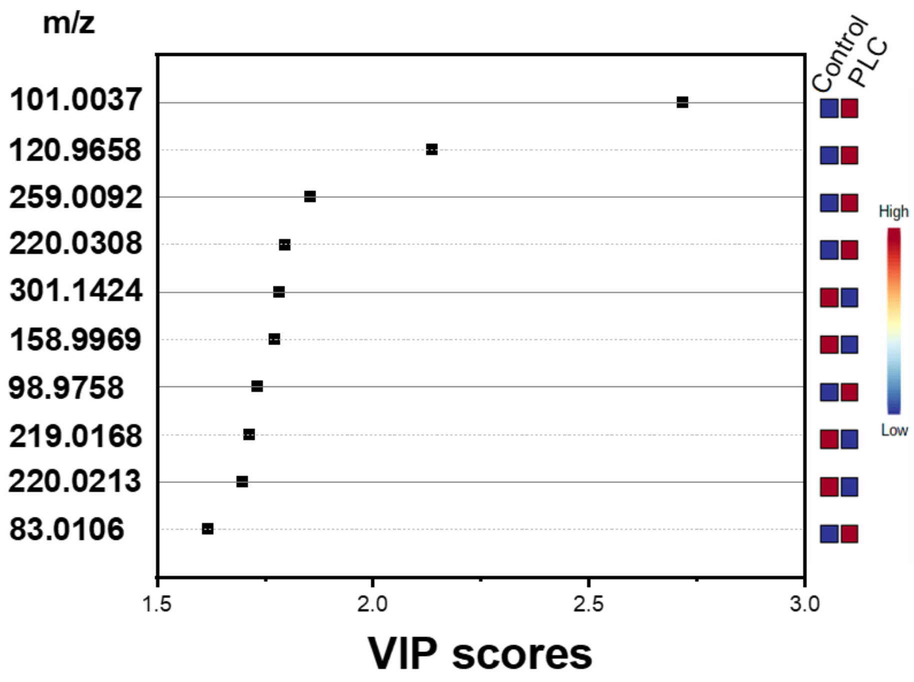

Fig. S12.

Significant features identified by OPLS-DA. The features are ranked by variable importance on projection value.

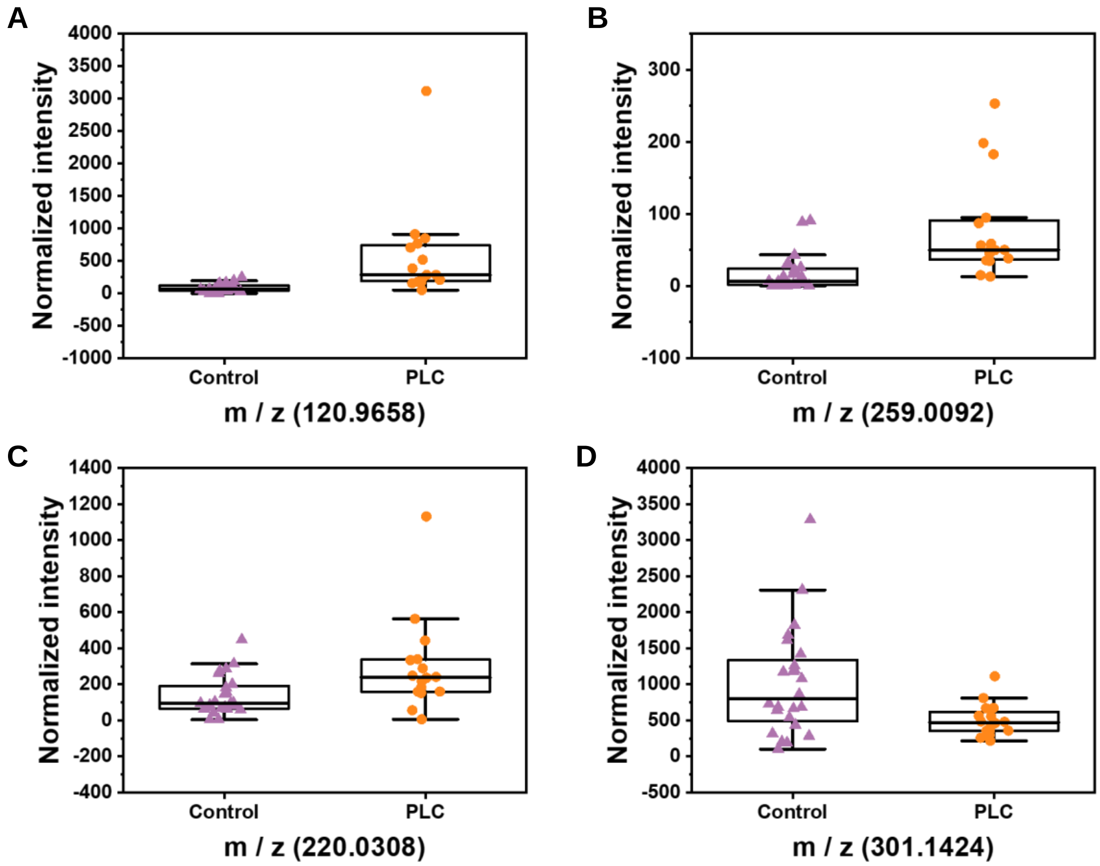

Fig. S13.

Comparison of m/z values used for distinguishing PLC from control in training dataset.

Table S1.
Information about analyte recovery.

Initial content (ng/mL)	Added content (ng/mL)	Contents after adding standard (ng/mL)	Recovery (%)
76.23	83.33	143.18	80.34
		149.34	87.73
		162.77	103.85

Table S2.
The specific m/z values and charges of detected HSA.

Name	m/z	Charge
Human Serum Albumin (HSA)	1517.83	44+
	1553.08	43+
	1592.83	42+
	1630.08	41+
	1672.33	40+
	1714.83	39+
	1757.08	38+
	1806.08	37+
	1856.67	36+

Table S3.
Information about the donors.

Characteristic	Age (mean± s.d.)	Gender (male/female)
PLC (n=24)	65±12	16/8
Control (n=38)	59±12	20/18

Table S4.

Detailed m/z values of volcano plot.

m/z value	Fold change (FC)	log_2(FC)	P value	FDR
120.9658	6.8012	2.7658	2.44E-08	1.71E-06
259.0092	4.4161	2.1428	6.43E-06	0.000225
101.0037	6.9635	2.7998	0.00141	0.003296
221.0496	2.3486	1.2318	0.00161	0.024161
220.0308	2.1712	1.1185	0.002215	0.024161
220.0213	0.36208	-1.4656	0.00257	0.024161
158.9969	0.138	-2.8573	0.002606	0.11581
301.1424	0.49661	-1.0098	0.002798	0.024161
165.0128	3.2359	1.6941	0.003106	0.024161
98.9758	3.4659	1.7932	0.004776	0.030394
219.0168	0.40724	-1.296	0.006692	0.039036
414.2681	0.40496	-1.3042	0.008623	0.043477
262.9800	2.9222	1.5471	0.008695	0.043477
104.9920	3.0721	1.6192	0.011331	0.052876
244.2624	0.46113	-1.1167	0.012703	0.055577
291.5813	0.19585	-2.3522	0.033087	0.024161
201.0468	0.1974	-2.3408	0.047069	0.1569

Table S5.

Detailed m/z values of cis diols used in building models.

m/z	VIP rank in OPLS-DA	Fold change in Volcano plot	P value in training dataset	Q value in training dataset
101.0037	1	6.9635	0	0
120.9658	2	6.8012	0.04	0.12
158.9969	6	0.138	0.58	0.58
220.0308	4	2.1712	0.29	0.58
259.0092	3	4.4161	0.41	0.58
301.1424	5	0.49661	0.54	0.58

Table S6.

Detailed information of main cis-diols annotated from HMDB.

Name	Formula	Ion type	Measured m / z	Theoretical m / z	Relative error (ppm)
3,4-dihydroxy-5-(3-methylbut-2-en-1-yl)benzoic acid	$C_{12}H_{14}O_4$	M+H-H_2O	205.0853	205.0865	6
2,3-Dihydroxy-2,4-cyclopentadien-1-one	$C_5H_4O_3$	M+Na	135.0053	135.0053	0
benzene-1,2,3,5-tetrol<br><br>benzene-1,2,3,4-tetrol	C_6H_6O4	M+Na	165.0142	165.0158	10
Dihydrodeoxy-8-epiaustdiol	$C_{12}H_{14}O_4$	M+H-H_2O	205.0853	205.0865	6
Chlorphenesin	$C_9H_{11}ClO_3$	M+K	241.0006	241.0028	9
3-Chloro-1-(4-hydroxy-3-methoxyphenyl)-1,2-propanediol	$C_{10}H_{13}ClO_4$	M+Na	255.0398	255.0395	1
Adenosine	$C_{10}H_{13}N_5O_4$	M+H	268.1056	268.1040	6
2,4-Dihydroxychalcone	$C_{15}H_{12}O_3$	M+K	279.04	279.0418	6
2-(4-hydroxy-3-methylbut-2-en-1-yl)-4-(3-methylbut-2-en-1-yl)benzene-1,3,5-triol<br><br>2-[(3,3-dimethyloxiran-2-yl)methyl]-4-(3-methylbut-2-en-1-yl)benzene-1,3,5-triol<br><br>bis(3-methylbut-2-en-1-yl)benzene-1,2,3,5-tetrol<br><br>2-{[3-methyl-3-(4-methylpent-3-en-1-yl)oxiran-2-yl]methyl}benzene-1,3,5-triol<br><br>4-(3,7-dimethylocta-2,6-dien-1-yl)benzene-1,2,3,5-tetrol<br><br>2-(5-hydroxy-3,7-dimethylocta-2,6-dien-1-yl)benzene-1,3,5-triol<br><br>2-(4-hydroxy-3,7-dimethylocta-2,6-dien-1-yl)benzene-1,3,5-triol	$C_{16}H_{22}O_4$	M+H<br><br>M+Na<br><br>M+K	279.1582<br><br>301.14<br><br>317.1157	279.1591<br><br>301.1410<br><br>317.1150	3<br><br>3<br><br>2

2-[5-(3,3-dimethyloxiran-2-yl)-3-methylpent-2-en-1-yl]benzene-1,3,5-triol<br><br>2-[(2Z)-4-hydroxy-3-(4-methylpent-3-en-1-yl)but-2-en-1-yl]benzene-1,3,5-triol<br><br>2-[(6E)-8-hydroxy-3,7-dimethylocta-2,6-dien-1-yl]benzene-1,3,5-triol<br><br>2-(8-hydroxy-3,7-dimethylocta-2,6-dien-1-yl)benzene-1,3,5-triol					
Uridine<br><br>Pseudouridine	$C_9H_{12}N_2O_6$	M+K	283.0328	283.0327	0
Guanosine	$C_{10}H_{13}N_5O_5$	M+H	284.1007	284.0989	6
Ribothymidine<br><br>Imidazoleacetic acid riboside<br><br>3-Methyluridine	$C_{10}H_{14}N_2O_6$	M+K	297.0483	297.0483	0
Ellagic acid	$C_{14}H_6O_8$	M+H	303.0105	303.0135	10
2,4-Dihydroxy-1,4-benzoxazin-3-one glucuronide	$C_{14}H_{15}NO_{10}$	M+H-H_2O	340.062	340.0669	14
{3-[2-(3,4-dihydroxy-5-methoxyphenyl)ethyl]phenyl}oxidanesulfonic acid<br><br>{3-[2-(2,3-dihydroxy-5-methoxyphenyl)ethyl]phenyl}oxidanesulfonic acid	$C_{15}H_{16}O_7S$	M+H	341.0677	341.0690	14
MG(16:0/0:0/0:0)<br><br>MG(i-16:0/0:0/0:0)	$C_{19}H_{38}O_4$	M+Na	353.2652	353.2662	3

Reference

1. Y. Li, M. Bouza, C. Wu, H. Guo, D. Huang, G. Doron, J. S. Temenoff, A. A. Stecenko, Z. L. Wang and F. M. Fernández, *Nat. Commun.* 2020, **11**, 5625.
2. H. Tsugawa, T. Cajka, T. Kind, Y. Ma, B. Higgins, K. Ikeda, M. Kanazawa, J. VanderGheynst, O. Fiehn and M. Arita, *Nat. Methods* 2015, **12**, 523-526.
3. C. Guijas, J. R. Montenegro-Burke, X. Domingo-Almenara, A. Palermo, B. Warth, G. Hermann, G. Koellensperger, T. Huan, W. Uritboonthai, A. E. Aisporna, D. W. Wolan, M. E. Spilker, H. P. Benton and G. Siuzdak, *Anal. Chem.* 2018, **90**, 3156-3164.
4. D. S. Wishart, D. Tzur, C. Knox, R. Eisner, A. C. Guo, N. Young, D. Cheng, K. Jewell, D. Arndt, S. Sawhney, C. Fung, L. Nikolai, M. Lewis, M.-A. Coutouly, I. Forsythe, P. Tang, S. Shrivastava, K. Jeroncic, et al. *Nucleic Acids Res.* 2007, **35**, D521-D526.
5. J. Xia and D. S. Wishart, *Nat. Protoc.* 2011, **6**, 743-760.
6. J. Xia, R. Mandal, I. V. Sinelnikov, D. Broadhurst and D. S. Wishart, *Nucleic Acids Res.* 2012, **40**, W127-W133.
7. J. Chong and J. Xia, *Bioinformatics*, 2018, **34**, 4313-4314.
8. CLSI. Protection of Laboratory Workers from Occupationally Acquired Infections; Approved Guideline– Third Edition. CLSI. document M29-A3 (ISBN 1-56238-567-4).
